# Sex Manipulation Technologies Progress in Livestock: A Review

**DOI:** 10.3389/fvets.2020.00481

**Published:** 2020-08-14

**Authors:** Yanshe Xie, Zhiqian Xu, Zhenfang Wu, Linjun Hong

**Affiliations:** ^1^Guangdong Provincial Key Laboratory of Agro-Animal Genomics and Molecular Breeding, National Engineering Research Center for Breeding Swine Industry, College of Animal Science, South China Agricultural University, Guangzhou, China; ^2^Lingnan Guangdong Laboratory of Modern Agriculture, Guangzhou, China

**Keywords:** sex manipulation, sperm separation, female reproductive tract, embryo sexing, livestock

## Abstract

Sex manipulation technologies allow predetermination of the sex of animal offspring by altering the normal reproductive process. In livestock production, the difference in type and gender can translate into significant economic benefits, including alleviation of severe food shortages. In livestock, however, the commercial application of sex manipulation technologies is currently available for cattle only. In this review, we described the brief history of sex manipulation, and the research progresses of common methods used in sex manipulation thus far. Information presented in this review can inform future studies on expanding the scope and use of sex manipulation technologies in livestock.

## Introduction

By 2050, the world's population is expected to increase by 9.6 billion, and the demand for livestock products, by 70 percent (1). Most of the population growth is however expected to occur in developing countries, where currently most croplands in production face challenges in coping with urbanization, biofuel production, and climate change (2). Therefore, to meet the forecasted global food demand, utilizing modern biotechnologies to promote sustainable production of livestock will be essential. Sex manipulation technologies are biotechnologies that can selectively predetermine the sex of animal offspring by intervening the normal reproductive process, and besides playing an important role in solving food shortage problem, it can address issues related to animal welfare such as castration. In this review, our objective was to provide suggestions for future research to improve sex manipulation technologies in livestock.

## The Origin and Development of Sex Manipulation

In ancient times, boys were also preferred to girls since besides being more likely to appease the wrath of the gods, they were regarded to be a strong support for the elderly (3). The preference for producing male offspring therefore promoted sex manipulation research (4).

Theories of what determined the sex of an individual transited from myths to science in the 17th century. In 1651,William Harvey compared the reproductive organs of several animals and stated that “all animals whatsoever, even viviparous creatures, man himself, are all engendered from an egg” (5). Later on, in the 1670's, AntonieVanLeeuwenhoek discovered a microscope which he used to observe sperm cells in human and dog seminal fluid. This discovery became a turning point in the research of sex manipulation (6).

In 1905 while studying the mealworm beetle *Tenebrio molitor*, Nettie Maria Stevens found that unlike in females, one chromosome was smaller than others in males. She concluded that the shorter chromosome was the “Y” chromosome, and was responsible for sex determination alongside the larger chromosome, which she named chromosome “X” (7).

By the end of the 1950's, the Y chromosome's role in determining the male sex was undisputed. Thus, over the next few decades, efforts were geared toward identifying a single testis-determining factor (*TDF*) in humans. In 1990, researchers found a 35-kb region from Y chromosome in four masculinized XX patients. Southern blotting revealed that the Y-fragment was the likely carrier of the TDF gene (8). Sequencing showed that an open reading frame (ORF) encoded a single-exon gene named the “sex-determining” region of the “Y chromosome” (*SRY* in humans) (9). Further, researchers found that *SRY* up-regulated the expression of *SOX9* in somatic (pre-Sertoli) cells of the genital ridge (10). Besides the relationship with autosomal sex reversal and Campomelic Dysplasia (CD) in human (11), *SOX9* can activate the expression of genes required for Sertoli cell differentiation [e.g., *FGF9* (12)], causing the re-expression of genes associated with ovarian development [e.g., *WNT4* (13)]. Importantly, *SOX9* also promotes its own expression, thereby bypassing the testicular differentiation that requires sustained *SRY* expression (14). Therefore, the discovery of *SRY* on the Y chromosome was a major breakthrough in the mammalian sex determination theory and promoted the development of sex manipulation technologies by providing a theoretical basis. Thereafter, the research on sex manipulation technologies gained traction with various studies seeking to enhance the respective existing technologies. Herein, we discussed the advances in sex manipulation technologies in animals with a focus of providing an informative insight for future development.

## Sex Selection Strategies During Spermatogenesis

The independent orientation of homologous chromosome pairs along the metaphase plate during metaphase I of meiosis and the subsequent separation of homologs and sister chromatids during anaphase I of meiosis allow an independent distribution of X or Y chromosomes to each secondary spermatocyte (15). During meiosis, some genes encode proteins with essential roles in structures or functions specific to spermatogenic cells, are expressed in developmentally regulated patterns, and are transcribed only in, or produce mRNAs unique to, spermatogenic cells (16). Therefore, the expression of these genes can regulate the ultimate meiotic stage-specific protein expression and change the ratio of X- and Y-bearing sperm during spermatogenesis.

In recent years, with the development of molecular biology and cell biology, there were more and more sex-determining genes being identified, including *Sry* (17, 18), *Amh* (19), *Sox* family (10, 20, 21), *Dmrt1* (22), *Fgf9* (23), *Gata4* (24), etc., which are associated with male sex determination, and *Wnt4* (25–27), *Dax1* (28), *Rspo1* (29, 30), *Foxl2* (27, 31–33) associated with female sex determination. Presently, gene edit and RNA interference targeting these sex-determining genes are the main methods often used to bias the ratio of X- and Y-bearing sperm during spermatogenesis.

The emergence of gene editors such as zinc finger nucleases (ZFN), transcription-activator-like endonucleases (TALENs), and the clustered regularly interspaced short palindromic repeats/Cas endonucleases (CRISPR/Cas) system has offered great potential in the selection of sex at the gene level (34).

Successful ZFN-mediated disruption of a sex-determining gene in rainbow trout (35) and the generation of *Sry* knockout mouse via oocyte injection using TALEN (36) have since been reported. However, standard molecular biology laboratories cannot afford the demand of ZFN molecules because most ZFN molecules are commercial. Although TALENs are more effective than ZFNs, the design of TALEN is complicated and time-consuming due to the high amounts of plasmids involved. Moreover, the specificity of TALEN to target the expected DNA sequences remains unconfirmed. The CRISPR/Cas system is superior to ZFNs and TALENs due to its ease of use and cost-effectiveness, provided a molecular biology laboratory has access to CRISPR kits. Besides, the CRISPR/Cas system can identify and induce mutation on target loci with high specificity (37). Although these gene editors cannot be used in livestock production presently, the CRISPR/Cas system is the most suitable technique for sex selection among them.

The CRISPR/Cas system is usually introduced into zygotes via microinjection (38, 39) or combined with somatic cell nuclear transfer or surgical transfer to generate offspring (40). Recently, researchers have biased the sex ratio of populations of different organisms such as plants, insects, crustacean, and fish, at the gene level (41). The CRISPR/Cas system has been used to control agricultural pest through female-elimination approaches that give rise to more males in an insect population. Such a rise can either result in a population decrease or eliminate the population occasioned by the lack of females (42).

A recent study in mice described a new strategy to bias the sex ratio of the offspring. Three CRISPRguide RNAs targeting three autosomal genes which are essential for early development in mouse were encoded on murine Y paternal line, while the CRISPR-Cas9 enzyme was encoded into the maternal line. After fertilization, both the Y-encoded guide RNAs from the paternal sperm and the Cas9 protein from the maternal egg targets resulted in self-destructing males (41). The high homology of the Sp1-binding site between humans and rabbits makes the latter a suitable animal model for studying the clinical male-to-female sex reversal syndrome in humans. The first rabbit hermaphroditism model was recently generated using the CRISPR/Cas9 system. Ten potential off-target sites were analyzed by Sanger sequencing and the T7E1 assay to determine whether off-target effects occurred in the *SRY* mosaic rabbit. To determine whether chimeric mutations in the *SRY* gene induced hermaphroditism, the internal genitalia of *SRY*-mutant chimeric rabbits were also analyzed. The generation of the model provided a platform to understand the pathogenesis of hermaphroditism and identify novel therapies for human clinical hermaphroditism with male-to-female sex reversal syndrome (43).

Gene editing technology requires highly skilled personnel, and the lack of a clear role of the sex-determining gene in livestock sex determination hinders its application in this field (34). However, the optimization of the existing gene editors, the development of new gene editors, and continued research on the sex-determining gene will aid in breaking the barrier. However, there has been a long running debate over whether bio-technologies should be applied into food production. Although, many of GM (genetically modified) organisms are already deemed safe, the safety of GM food has been questioned by the general public ever since their introduction in the late 90's (44, 45), which hindered the development of bio-technologies in food production. Therefore, government may play an important role in driving the bio-technologies in food production forward by setting stricter food quality standards, improving legal regulation on GM food, and choosing a mandatory GM food labeling system to protect the consumer's right to know. Changing the sex ratio of offspring at the gene level cannot only promote the development of livestock production but also provide a therapeutic biological model for human diseases.

RNA interference (RNAi) is a biological process that uses small interfering RNA (siRNA) to induce sequence-specific gene silencing through mRNA degradation (46). Since its first use in mammalian cells in 2001, RNAi technology has successfully been implemented in mammalian gene silencing systems (47). Genes such as *Sry, zfy*, and *zfx* that are known to affect sex determination during spermatogenesis could be silenced through RNAi to influence the sex ratio of animal offspring.

Research has demonstrated that injecting RNAi vectors into mouse testis can skew the sex-ratio of offspring (48). In a separate study, knocking out *Sry* mRNA by direct injection of siRNA into pregnant mice through the tail vein generated mouse embryos with feminized gonads (49).

The mechanisms of these sex-determining genes in spermatogenesis and sex determination are uncertain, and very little is known on whether these genes could be knocked-down using RNAi technology or the CRISPR/Cas9 system. This is because the expression level of mRNA is influenced by several factors including non-specific creation, the activation of innate immune response, and off-target effects. In addition, transfection protocols yield low efficiency for primary cells, while suspension requires optimization for different cell types. Besides, poor bioavailability, rapid hydrolysis, and the inability to cross biological barriers such as the blood-brain barrier are some of the major concerns that hinder RNAi adoption. However, it has been established that exosomes can transfer siRNAs with high target specificity and without any immunologic reactions as a natural vehicle, thereby providing a new method for delivering of siRNAs into the cells (50).

Primarily, RNAi is a simple method that has had a significant effect on the expression of the sex-determining gene. Future studies should therefore explore the mechanisms of sex-determining genes in spermatogenesis and sex determination, improve transfection efficiency, and reduce electropuncture damage. Besides, using exosome as a vehicle for targeted delivery has good prospects for the future.

## Sperm-Separating Technologies in Sex Manipulation

Several methods have been previously used for separating X- and Y-bearing sperm. The initial sperm sexing procedures designed for sex determination involved DNA probes (51, 52), albumin gradients (53, 54), Percoll gradients (55, 56), the Sephadex gel-filtration method (57–59), and “swim-up” procedures (60, 61). However, the efficiency of these methods in distorting the sex ratio of livestock offspring has been unconvincing (62). Additionally, all these methods can damage sperm motility. Therefore, flow cytometry and immunology sexing are the main sperm separating technologies presently used in livestock production at present.

### Flow Cytometry in Sex Manipulation

Johson et al. was the first to use flow cytometry to separate sperm according to the difference of DNA content in the head of X- and Y-bearing sperm (the DNA content of X-bearing sperm is 3–4% higher than that of Y-bearing sperm) in 1986 (63, 64). Since then, flow cytometry-based sperm sexing has produced millions of pre-sexed offspring in different species including, rabbits, pigs, cattle, sheep, horses, dogs, cats, etc.

Generally, contemporary sperm-sorting procedures yield a skewed offspring sex ratio of between 85 and 95% accuracy (65). Flow cytometry has since reached commercial application in cattle (66), and the quality of sperm for use in a flow sorter is dependent on the species. Therefore, the sorting index of different species is calculated by multiplying the percentage of the difference in DNA content of the X- or Y-bearing sperm by the area of the flat profile of the sperm head. The sorting index indicates that bull and boar sperm are well-suited for separation in a flow sorter (133 and 115, respectively) (67). However, use of flow cytometry to sort sperm is minimal in pig production. This could be a result of boar sperm being more susceptible to the influence of flow cytometry since the sorting process damages the boar sperm membrane, which leads to the decline in its vitality, fertilization ability, storage ability, and subsequent processing ability (68, 69). In addition, the sperm used for artificial insemination per pregnancy in pig production is often significantly higher than in cattle production (70, 71).

Initial studies focused on identifying the steps in flow cytometry that caused sperm damage. It was earlier thought that optimization of sperm dilution rate (72–74), as well as changing the type of dyes and laser power (75–77) could reduce sperm damage. However, these methods could not significantly increase sex-sorted sperm motility. This prompted other researches to focus on improving hardware, optics, electronics, and handling protocols before flow cytometry such as reducing the sheath pressure of the sorter from 50 to 40 psi (78), implementation of pulsed lasers (79), or transportation of red deep sperm at high sperm concentration without staining before sorting (80). Although these improvements increased the motility of sperm after separation, subsequent optimization is limited by current mechanical technology. Besides, improving the production speed and sperm quality remains a significant challenge, thereby shifting attention to related technology.

In a study that aimed to describe the effects of flow cytometry on the structure of the glycocalyx of bovine sperm by using lectin histochemistry, both flow cytometry and capacitation had similar effects on bovine sperm, which may account for the low conception rates observed after flow cytometry (81). Another study that combined AA-2G supplementation and deep insemination to improve the fertility of sex-sorted sperm from Cashmere goats revealed a substantial protective effect on the quality and functions of sperm (82). Although some researchers prefer artificial insemination technology, laparoscopic insemination (LI) at the uterine horns can achieve a high fertilization rate with a low sex-sorted sperm dose (five times less than intrauterine insemination) (83). However, improvements such as the outputs of the flow sorters, aspects related to the synchronization protocols, the time of ovulation, and single LI at the uterine horns deserve to be optimized in future studies (84).

In conclusion, flow cytometry is the most used and mature technology for sperm sexing in livestock production, with a separation accuracy of above 90%. However, due to the high sperm requirement and susceptibility of other species, this strategy has only reached commercial application in cattle production. Future studies should therefore focus on reducing sperm damage caused by flow cytometry, develop related technologies, and optimize artificial insemination technology.

### Immunology Sexing in Sex Manipulation

Immunology sexing is achieved through the identification and separation of sex-related proteins. This is mainly done by detecting the H-Y antigen on the plasma membrane of Y-bearing sperm by using the H-Y antibody. Thus, far, sperm from many species has been successfully separated including mice (85), rabbits (86), and sheep (87). In addition, because of the simplicity, rapidity, accuracy, and reasonable price of this method, it can be used to verify the purity of sorted X- and Y-bearing sperm (88).

A recent study successfully separated sperm from mice by combining the immunology sexing and swim-up methods, thereby providing a strategy for immunology sexing. In 2019, three Japanese scientists found that Toll-like receptor 7/8, which is encoded by X chromosome, could influence sperm motility. The motility of X sperm reduces when incubated with R848 (a synthetic agonist which can bind both TRL8 and TLR7) as a result of Toll-like receptor 7/8 binding R848. However, the motility of the Y sperm is not affected. Thus, the sperm is selectively separated by using the swim-up method, in which Y sperm with high motility swims to the upper layer of the medium, while the X sperm sinks to the lower layer of the medium. Besides, R848 had no effect on the acrosome of both X sperm and Y sperm, indicating that sperm fertility is not affected even after incubating with R848. In addition, the motility of X sperm returns to normal once R848 has been washed off. The sperm separated by this method was later used for *in vitro* fertilization and embryo transfer. In the results, upstream sperm produced 77 blastocyst embryos, among which 68 were XY embryos and 9 were XX embryos, while downstream sperm produced 83 blastocyst embryos, among which 58 were XY embryos and 25 were XX embryos (89).

However, most immunology sexing strategies affect the motility of sperm and reduce the conception rate due to the long processing time. Although this strategy is presently at the laboratory stage, advances in molecular biology may provide more breakthroughs. Therefore, more efforts should be concerted on the discovery of new sex-related proteins and the blending of other strategies to reduce sperm damage.

## Sex Manipulation Within the Female Reproductive Tract

Millions or billions of sperm are deposited into the reproductive tract after mating, but only a few sperm reach the ampulla or the site of fertilization and only one fertilizes an oocyte. The journey that sperm take to reach an oocyte is long and perilous. The female environment contributes toward this successful transit by providing a vehicle for sperm transport, aiding the removal of dead sperm and other pathogens and applying strict selection pressures to ensure only those cells with the highest quality reach the site of fertilization. Besides, in many locations of the female reproductive tract, sperm interact with the epithelium and the luminal fluid, which can affect sperm motility and function (90, 91). Although we know little about this complex interaction between sperm and female environment, many researches sought to reveal the impact of the mammalian female on the sex of her posterity.

By introducing X- or Y-bearing sperm into two separate oviducts of single female pigs using bilateral laparoscopic insemination, researchers demonstrated that the oviduct can screen sperm by modifying the oviduct environment (92). Besides, research has shown that both time of ovulation and maturational state of oocyte can influence the sex ratio of animal offspring (93, 94). This led to the investigation of the effect of follicular fluid composition on the sex of an embryo, which was confirmed in subsequent studies (95, 96). In addition, various studies have reported the impact of pH on sperm motility, viability, and capacitation (97). However, pH did not influence the X- or Y-bearing sperm of bovine *in vitro* (98). Therefore, extensive studies are needed to examine the effect of external pH on X- or Y-bearing sperm. We speculate that pH might affect X- or Y-bearing sperm by altering other physiochemical factors in the bovine oviduct. Furthermore, there have been several papers suggesting the effect of several glycoproteins and factors from the female reproductive tract on sperm survival including L-selectin (99), PMCA4 (100), fibronectin (101), SBG (102), etc. Hence, manipulating the sex of the offspring by regulating the expression level of these glycoproteins and factors is possible and could be a new direction for future research.

Above all, although sex manipulation in the female reproductive tract is the most parsimonious strategy proposed thus far, it has the potential of resolving the formerly puzzling and seemingly contradictory findings. With continued research, a better understanding of female influence in sex determination can be attained and could have a huge impact on existing sex manipulation strategies.

## Embryo Sexing in Sex Manipulation

Since artificial insemination and embryo transfer technology were implemented in commercial field, research on finding embryo sexing method has become a point of interest for modern researchers (103). Obstetric ultrasonography is the most common imaging technique in veterinary reproduction including: confirming pregnancy, identifying congenital fetal defects, defining fetal sex, etc., because it is simple, reliable, and non-invasive (104, 105). However, the sex of mammalian fetus cannot be performed by ultrasonographic examination until several weeks after pregnancy [e.g., 55–85 days in cattle (106), 59–68 days in horse (107), 46–55 days in goat (108)], which does not meet the requirements of embryo transfer as the main stage at which embryo transfer is performed is the cleavage stage (day 2–4 after fertilization) or the blastocyst stage (day 5 or 6 fertilization) (109). Thus, a large number of techniques for genetic sex identification of early embryo in mammals have since been developed. Before Barr body formation (110) or detection of H-Y antigen (111, 112), methods used were based on cytogenetic analysis (113, 114) and measurement of level of X-linked enzymes (glucose-6-phosphatase dehydrogenase, G6PD). However, due to the high embryo requirement, low accuracy, and the potential of embryo injuries, these methods were replaced by molecular detection.

The blastomere of mammalian embryos is omnipotent and can develop into a complete animal. Thus, each blastomere contains all the genes required for the development of an animal, including genes involved in sex determination such as *Amg* (115), *Zfy* (116), and *Sry* (117). Molecular biologic detection techniques, which include using nucleic acid probe, fluorescence *in situ* hybridization (FISH), polymerase chain reaction (PCR), and loop-mediated isothermal amplification (LAMP), can therefore be used to identify the male-specific gene.

Among nucleic acid probes, isotopic labeled probes have high accuracy but require a lot of embryo cells and time, while biotin-labeled probes require little time to detect but can only separate few embryos (118). FISH, on the other hand, has high accuracy and rapidity and can display a variety of colors due to the emergence of multi-color fluorescence *in situ* hybridization. FISH is however expensive and complex to operate (119), thereby leaving PCR and LAMP as the mainstream methods for embryo sexing in sex manipulation presently.

PCR is widely used, and besides its ability to analyze the phenotypes based on amplification products of sex-specific genes, it can verify the purity of sorted X- and Y-bearing sperm statistically. To identify the sex of embryos (120) through PCR, a pair of previously developed sex-specific primers was used. In 1991, this method successfully identified the sex of cattle and sheep embryos for the first time (121). However, since this method only amplified a pair of sex-specific primers, lack of amplification would correspond to a negative result. This method was therefore replaced by multiplex PCR in which a pair of sex-specific primers with at least one pair of internal primers that amplify house-keeping genes (122) was designed and utilized. However, due to the large number of cells required in the conventional PCR method and the limited sensitivity of one amplification, the method was prone to errors. Therefore, nested PCR and multiplex PCR were later combined to improve the accuracy of detection results. Besides increasing the amount of amplification product and the test's accuracy, the combination reduced the number of required cells to 3–8. In addition, conventional PCR has some disadvantages such as a complicated identification process and long identification time. Until 1992, Cha et al. adopted two-temperature gradient PCR to obtain the ideal amplification results. This proved that DNA polymerase could rapidly catalyze a complete amplification process from annealing to degeneration without extension steps when the fragment amplified is small (<200 bp). Not only did this retain the advantages (high sensitivity, high accuracy, low embryonic damage) of conventional PCR, but it also simplified the conventional PCR and shortened the entire process from 2–4 h to 1 h (123).

Presently, PCR can be used for further analyses, such as genotyping and molecular diagnosis in preimplantation embryos besides embryo sexing (124). Moreover, it is feasible for automation and high-throughput sex-typing (125). Though not suitable for the sex manipulation of twin pregnancies (126), research has demonstrated that sex manipulation using the DNA of free fetal cells in maternal plasma is efficient for both *SRY* and *AMEL* gene sequences.

Loop-mediated isothermal amplification (LAMP) is a method of DNA amplification that was developed by Eiken Chemical Co. Ltd. in 2000 and works under isothermal conditions. This method of DNA amplification is highly specific, efficient, and rapid (127). Besides, gene amplification and detection can be completed in one step, and amplification could be up to 10^9^-10^10^ times in 15–60 min. Since the detection of all target gene sequences can only be determined by either the presence or absence of amplification products, LAMP is considered to be highly specific. Recently, LAMP primers with polyethylenimine (PEI) for precipitation were optimized to improve sensitivity, and multiplex LAMP was used to improve accuracy (128). The first LAMP assay for the detection of the *SRY* gene was performed using DNA extracted from 15 blood samples from pregnant women at 8 weeks (129).

Although PCR and LAMP have made significant progress in embryo sexing owing to their high sensitivity, the impact of the pollution in the external environment on the accuracy of the results remains inevitable. In addition, these two methods are difficult to use in livestock production due to the large sample size and the poor transfer pregnancy rate of recent embryo transfer technology. Therefore, future research should focus on improving tolerance to external environment and the efficiency of these technologies. Besides, these technologies should be applied to identify the accuracy of other sex manipulation strategies, rather than as a sex manipulation strategy.

## Conclusion

Sex manipulation technologies are valuable biotechnologies that have the potential to revolutionize cattle production. With the development of molecular biology and cell biology, these emerging sex manipulation strategies significantly expand the ability to bias the sex ratio of animal offspring and would be instrumental in correcting clinical male-to-female sex reversal syndrome in humans. However, the knowledge of sex-determining mechanisms, whether sex-determining gene or female reproductive tract, is still very limited. Since different sex manipulation strategies have their individual advantages and disadvantages, the selection of a specific system is dependent on the expertise of the individual researcher rather than on the weaknesses of one of these strategies. Collectively, sex determination is a complex process that is influenced by a variety of internal and external factors. Further exploration of sex-determining mechanisms; continuous improvement of the accuracy, sensitivity, and economic benefits of sex manipulation technologies; and the establishment of a new a sex manipulation system that integrates knowledge from various fields can provide great impetus in livestock production and solve the problem of food shortage.

## Author Contributions

YX and ZX: conceived the idea, conducted the literature review, and drafted the manuscript. ZW and LH: project investigator, decided a review topic, and revised the manuscript. All the authors have read and approved the final version of the manuscript.

## Conflict of Interest

The authors declare that the research was conducted in the absence of any commercial or financial relationships that could be construed as a potential conflict of interest.

## References

[1] Stratonovitch P Semenov MA. Heat tolerance around flowering in wheat identified as a key trait for increased yield potential in Europe under climate change. J Exp Bot. (2015) 66:3599–609. 10.1093/jxb/erv070 25750425 PMC4463804

[2] Moda HM Filho WL Minhas A. Impacts of climate change on outdoor workers and their safety: some research priorities. Int J Environ Res Public Health. (2019) 16:3458. 10.3390/ijerph16183458 31533360 PMC6765781

[3] Mccartney ES Sex determination and sex control in antiquity. Am J Philol. (1922) 43:62–70. 10.2307/289330

[4] Stévant I Papaioannou MD Nef S. A brief history of sex determination. Mol Cell Endocrinol. (2018) 468:3–10. 10.1016/j.mce.2018.04.004 29635012

[5] Oppenheimer JM. Disputations touching the generation of animals. Essay review. Trans Stud Coll Phys Phila. (1982) 4:306–9. 6758233

[6] Ruestow EG. Images and ideas: leeuwenhoek's perception of the spermatozoa. J Hist Biol. (1983) 16:185–224. 10.1007/BF00124698 11611237

[7] Stevens NM Studies in Spermatogenesis. Washington, DC: Carnegie Institution of Washington (1905). p. 2.

[8] Palmer MS Sinclair AH Berta P Ellis NA Goodfellow PN Abbas NE. Genetic evidence that ZFY is not the testis-determining factor. Nature. (1989) 342:937–9. 10.1038/342937a0 2594087

[9] Sinclair AH Berta P Palmer MS Hawkins JR Griffiths BL Smith MJ. A gene from the human sex-determining region encodes a protein with homology to a conserved DNA-binding motif. Nature. (1990) 346:240–4. 10.1038/346240a0 1695712

[10] Sekido R Lovell-Badge R. Sex determination involves synergistic action of SRY and SF1 on a specific Sox9 enhancer. Nature. (2008) 453:930–4. 10.1038/nature06944 18454134

[11] Foster JW Dominguez-Steglich MA Guioli S Kwok C Weller PA Stevanović M. Campomelic dysplasia and autosomal sex reversal caused by mutations in an SRY-related gene. Nature. (1994) 372:525–30. 10.1038/372525a0 7990924

[12] Kim Y Kobayashi A Sekido R Dinapoli L Brennan J Chaboissier MC. Fgf9 and Wnt4 act as antagonistic signals to regulate mammalian sex determination. PLoS Biol. (2006) 4:e187. 10.1371/journal.pbio.0040187 16700629 PMC1463023

[13] Jameson SA Natarajan A Cool J Defalco T Maatouk DM Mork L. Temporal transcriptional profiling of somatic and germ cells reveals biased lineage priming of sexual fate in the fetal mouse gonad. PLoS Genet. (2012) 8:e1002575. 10.1371/journal.pgen.1002575 22438826 PMC3305395

[14] Koopman P Sinclair A Lovell-Badge R. Of sex and determination: marking 25 years of randy, the sex-reversed mouse. Development. (2016) 143:1633–7. 10.1242/dev.137372 27190031

[15] Monaghan F Corcos A. On the origins of the mendelian laws. J Hered. (1984) 75:67–9. 10.1093/oxfordjournals.jhered.a109868 6368675

[16] Eddy EM. Regulation of gene expression during spermatogenesis. Semin Cell Dev Biol. (1998) 9:451–7. 10.1006/scdb.1998.0201 9813192

[17] Zhao L Koopman P. SRY protein function in sex determination: thinking outside the box. Chromosome Res. (2012) 20:153–62. 10.1007/s10577-011-9256-x 22161124

[18] Gubbay J Collignon J Koopman P Capel B Economou A Munsterberg A. A gene mapping to the sex-determining region of the mouse Y chromosome is a member of a novel family of embryonically expressed genes. Nature. (1990) 346:245–50. 10.1038/346245a0 2374589

[19] Shahed A Young KA. Anti-mullerian hormone (AMH), inhibin-alpha, growth differentiation factor 9 (GDF9), and bone morphogenic protein-15 (BMP15) mRNA and protein are influenced by photoperiod-induced ovarian regression and recrudescence in siberian hamster ovaries. Mol Reprod Dev. (2013) 80:895–907. 10.1002/mrd.22215 23877969 PMC3835454

[20] Polanco JC Wilhelm D Davidson TL Knight D Koopman P. Sox10 gain-of-function causes XX sex reversal in mice: implications for human 22q-linked disorders of sex development. Hum Mol Genet. (2010) 19:506–16. 10.1093/hmg/ddp520 19933217

[21] Koopman P. Sex determination: a tale of two sox genes. Trends Genet. (2005) 21:367–70. 10.1016/j.tig.2005.05.006 15949865

[22] Matson CK Murphy MW Sarver AL Griswold MD Bardwell VJ Zarkower D. DMRT1 prevents female reprogramming in the postnatal mammalian testis. Nature. (2011) 476:101–4. 10.1038/nature10239 21775990 PMC3150961

[23] Bowles J Feng CW Spiller C Davidson TL Jackson A Koopman P. FGF9 suppresses meiosis and promotes male germ cell fate in mice. Dev Cell. (2010) 19:440–9. 10.1016/j.devcel.2010.08.010 20833365

[24] Hu YC Okumura LM Page DC. Gata4 is required for formation of the genital ridge in mice. PLoS Genet. (2013) 9:e1003629. 10.1371/journal.pgen.1003629 23874227 PMC3708810

[25] Jeays-Ward K Dandonneau M Swain A. Wnt4 is required for proper male as well as female sexual development. Dev Biol. (2004) 276:431–40. 10.1016/j.ydbio.2004.08.049 15581876

[26] Vainio S Heikkila M Kispert A Chin N Mcmahon AP. Female development in mammals is regulated by Wnt-4 signalling. Nature. (1999) 397:405–9. 10.1038/17068 9989404

[27] Ottolenghi C Pelosi E Tran J Colombino M Douglass E Nedorezov T. Loss of Wnt4 and Foxl2 leads to female-to-male sex reversal extending to germ cells. Hum Mol Genet. (2007) 16:2795–804. 10.1093/hmg/ddm235 17728319

[28] Swain A Narvaez V Burgoyne P Camerino G Lovell-Badge R. Dax1 antagonizes Sry action in mammalian sex determination. Nature. (1998) 391:761–7. 10.1038/35799 9486644

[29] Tomizuka K Horikoshi K Kitada R Sugawara Y Iba Y Kojima A. R-spondin1 plays an essential role in ovarian development through positively regulating Wnt-4 signaling. Hum Mol Genet. (2008) 17:1278–91. 10.1093/hmg/ddn036 18250097

[30] Chassot AA Ranc F Gregoire EP Roepers-Gajadien HL Taketo MM Camerino G. Activation of beta-catenin signaling by Rspo1 controls differentiation of the mammalian ovary. Hum Mol Genet. (2008) 17:1264–77. 10.1093/hmg/ddn016 18250098

[31] Takasawa K Kashimada K Pelosi E Takagi M Morio T Asahara H. FOXL2 transcriptionally represses Sf1 expression by antagonizing WT1 during ovarian development in mice. Faseb J. (2014) 28:2020–8. 10.1096/fj.13-246108 24451388 PMC3986836

[32] Crisponi L Deiana M Loi A Chiappe F Uda M Amati P. The putative forkhead transcription factor FOXL2 is mutated in blepharophimosis/ptosis/epicanthus inversus syndrome. Nat Genet. (2001) 27:159–66. 10.1038/84781 11175783

[33] Uhlenhaut NH Jakob S Anlag K Eisenberger T Sekido R Kress J. Somatic sex reprogramming of adult ovaries to testes by FOXL2 ablation. Cell. (2009) 139:1130–42. 10.1016/j.cell.2009.11.021 20005806

[34] Kurtz S Petersen B. Pre-determination of sex in pigs by application of CRISPR/Cas system for genome editing. Theriogenology. (2019) 137:67–74. 10.1016/j.theriogenology.2019.05.039 31208775

[35] Yano A Nicol B Jouanno E Guiguen Y. Heritable targeted inactivation of the rainbow trout (*Oncorhynchus mykiss*) master sex-determining gene using zinc-finger nucleases. Mar Biotechnol. (2014) 16:243–50. 10.1007/s10126-013-9546-8 24085607

[36] Kato T Miyata K Sonobe M Yamashita S Tamano M Miura K. Production of Sry knockout mouse using TALEN via oocyte injection. Sci Rep. (2013) 3:3136. 10.1038/srep03136 24190364 PMC3817445

[37] Fernandez A Josa S Montoliu L. A history of genome editing in mammals. Mamm Genome. (2017) 28:237–46. 10.1007/s00335-017-9699-2 28589393

[38] Hai T Teng F Guo R Li W Zhou Q. One-step generation of knockout pigs by zygote injection of CRISPR/Cas system. Cell Res. (2014) 24:372–5. 10.1038/cr.2014.11 24481528 PMC3945887

[39] Whitworth KM Benne JA Spate LD Murphy SL Samuel MS Murphy CN. Zygote injection of CRISPR/Cas9 RNA successfully modifies the target gene without delaying blastocyst development or altering the sex ratio in pigs. Transgenic Res. (2017) 26:97–107. 10.1007/s11248-016-9989-6 27744533 PMC5247313

[40] Hauschild J Petersen B Santiago Y Queisser AL Carnwath JW Lucas-Hahn A. Efficient generation of a biallelic knockout in pigs using zinc-finger nucleases. Proc Natl Acad Sci USA. (2011) 108:12013–7. 10.1073/pnas.1106422108 21730124 PMC3141985

[41] Yosef I Edry-Botzer L Globus R Shlomovitz I Munitz A Gerlic M. A genetic system for biasing the sex ratio in mice. Embo Rep. (2019) 20:e48269. 10.15252/embr.201948269 31267640 PMC6680165

[42] Tsoumani KT Meccariello A Mathiopoulos KD Papathanos PA. Developing CRISPR-based sex-ratio distorters for the genetic control of fruit fly pests: a how to manual. Arch Insect Biochem Physiol. (2020) 103:e21652. 10.1002/arch.21652 31845410

[43] Song Y Xu Y Liang M Zhang Y Chen M Deng J. CRISPR/Cas9-mediated mosaic mutation of SRY gene induces hermaphroditism in rabbits. Biosci Rep. (2018) 38:BSR20171490. 10.1042/BSR20171490 29439141 PMC5861328

[44] Bode L Contractor N Barile D Pohl N Prudden AR Boons GJ. Overcoming the limited availability of human milk oligosaccharides: challenges and opportunities for research and application. Nutr Rev. (2016) 74:635–44. 10.1093/nutrit/nuw025 27634978 PMC6281035

[45] Gabriels IJ Vergauwen L De Boevre M Van Dongen S Blust R De Saeger S. Optimizing the use of zebrafish feeding trials for the safety evaluation of genetically modified crops. Int J Mol Sci. (2019) 20:1472. 10.3390/ijms20061472 30909578 PMC6471220

[46] Han H. RNA interference to knock down gene expression. Methods Mol Biol. (2018) 1706:293–302. 10.1007/978-1-4939-7471-9_16 29423805 PMC6743327

[47] Elbashir SM Harborth J Lendeckel W Yalcin A Weber K Tuschl T. Duplexes of 21-nucleotide RNAs mediate RNA interference in cultured mammalian cells. Nature. (2001) 411:494–8. 10.1038/35078107 11373684

[48] Zhang Y Xi J Jia B Wang X Wang X Li C. RNAi as a tool to control the sex ratio of mouse offspring by interrupting Zfx/Zfy genes in the testis. Mamm Genome. (2017) 28:100–5. 10.1007/s00335-017-9682-y 28251288

[49] Wu N Yu AB Zhu HB Lin XK. Effective silencing of Sry gene with RNA interference in developing mouse embryos resulted in feminization of XY gonad. J Biomed Biotechnol. (2012) 2012:343891. 10.1155/2012/343891 22500086 PMC3303865

[50] Shahabipour F Barati N Johnston TP Derosa G Maffioli P Sahebkar A. Exosomes: nanoparticulate tools for RNA interference and drug delivery. J Cell Physiol. (2017) 232:1660–8. 10.1002/jcp.25766 28063231 PMC7166392

[51] Parrilla I Vazquez JM Oliver-Bonet M Navarro J Yelamos J Roca J. Fluorescence *in situ* hybridization in diluted and flow cytometrically sorted boar spermatozoa using specific DNA direct probes labelled by nick translation. Reproduction. (2003) 126:317–25. 10.1530/rep.0.1260317 12968939

[52] Kobayashi J Kohsaka T Sasada H Umezu M Sato E. Fluorescence *in situ* hybridization with Y chromosome-specific probe in decondensed bovine spermatozoa. Theriogenology. (1999) 52:1043–54. 10.1016/S0093-691X(99)00193-4 10735111

[53] Evans JM Douglas TA Renton JP. An attempt to separate fractions rich in human Y sperm. Nature. (1975) 253:352–4. 10.1038/253352a0 1110779

[54] Ericsson RJ Langevin CN Nishino M. Isolation of fractions rich in human Y sperm. Nature. (1973) 246:421–4. 10.1038/246421a0 4587152

[55] Resende MV Bezerra MB Perecin F Almeida AO Lucio AC De Lima VF Separation of X-bearing bovine sperm by centrifugation in continuous percoll and optiprep density gradient: effect in sperm viability and *in vitro* embryo production. Ciencia Anim Brasileira. (2009) 2:581–7.

[56] Aleahmad F Gourabi H Zeinali B Kazemi A S Baharvand H. Separation of X- and Y-bearing human spermatozoa by sperm isolation medium gradients evaluated by FISH. Reprod Biomed Online. (2009) 18:475–8. 10.1016/S1472-6483(10)60122-8 19400987

[57] Lobel SM Pomponio RJ Mutter GL. The sex ratio of normal and manipulated human sperm quantitated by the polymerase chain reaction. Fertil Steril. (1993) 59:387–92. 10.1016/S0015-0282(16)55682-9 8425636

[58] Beckett TA Martin RH Hoar DI. Assessment of the sephadex technique for selection of X-bearing human sperm by analysis of sperm chromosomes, deoxyribonucleic acid and Y-bodies. Fertil Steril. (1989) 52:829–35. 10.1016/S0015-0282(16)53048-9 2478397

[59] Steeno O Adimoelja A Steeno J. Separation of X- and Y-bearing human spermatozoa with the sephadex gel-filtration method. Andrologia. (1975) 7:95–7. 10.1111/j.1439-0272.1975.tb01234.x 1190509

[60] Hossain A Islam MM Naznin F Ferdousi RN Bari FY Juyena NS Quality of ram spermatozoa separated with modified swim up method. Bangladesh Vet. (2018) 33:62–70. 10.3329/bvet.v33i2.36459

[61] Repalle D Chittawar PB Bhandari S Joshi G Paranjape M Joshi C. Does centrifugation and semen processing with swim up at 37 degrees C yield sperm with better DNA integrity compared to centrifugation and processing at room temperature? J Hum Reprod Sci. (2013) 6:23–6. 10.4103/0974-1208.112375 23869146 PMC3713571

[62] Espinosa-Cervantes R Córdova-Izquierdo A. Sexing sperm of domestic animals. Trop Anim Health Pro. (2012) 45:1–8. 10.1007/s11250-012-0215-0 22829354

[63] Johnson LA Flook JP Look MV. Flow cytometry of X and Y chromosome-bearing sperm for DNA using an improved preparation method and staining with hoechst 33342. Gamete Res. (1987) 17:203–12. 10.1002/mrd.1120170303 3507347

[64] Johnson LA Clarke RN. Flow sorting of X and Y chromosome-bearing mammalian sperm: activation and pronuclear development of sorted bull, boar, and ram sperm microinjected into hamster oocytes. Gamete Res. (1988) 21:335–43. 10.1002/mrd.1120210402 3220427

[65] de Graaf SP Beilby KH Underwood SL Evans G Maxwell WMC. Sperm sexing in sheep and cattle: the exception and the rule. Theriogenology. (2009) 71:89–97. 10.1016/j.theriogenology.2008.09.014 18977523

[66] Vazquez JM Roca J Gil MA Cuello C Parrilla I Caballero I. Low-dose insemination in pigs: problems and possibilities. Reprod Domest Anim. (2008) 43:347–54. 10.1111/j.1439-0531.2008.01183.x 18638145

[67] Garner DL. Flow cytometric sexing of mammalian sperm. Theriogenology. (2006) 65:943–57. 10.1016/j.theriogenology.2005.09.009 16242764

[68] García EM Vázquez JM Parrilla I Calvete JJ Sanz L Caballero I. Improving the fertilizing ability of sex sorted boar spermatozoa. Theriogenology. (2007) 68:771–8. 10.1016/j.theriogenology.2007.06.006 17662382

[69] Maxwell WM Johnson LA. Physiology of spermatozoa at high dilution rates: the influence of seminal plasma. Theriogenology. (1999) 52:1353–62. 10.1016/S0093-691X(99)00222-8 10735081

[70] Bathgate R. Functional integrity of sex-sorted, frozen-thawed boar sperm and its potential for artificial insemination. Theriogenology. (2008) 70:1234–41. 10.1016/j.theriogenology.2008.06.009 18640713

[71] Vazquez JM Parrilla I Roca J Gil MA Cuello C Vazquez JL. Sex-sorting sperm by flow cytometry in pigs: issues and perspectives. Theriogenology. (2009) 71:80–8. 10.1016/j.theriogenology.2008.09.044 18977521

[72] Maxwell WMC Long CR Johnson LA Dobrinsky JR Welch GR Viability and membrane integrity of spermatozoa after dilution and cytometric sorting in the presence or absence of seminal plasma. Reprod Fertil Dev. (1997) 8:1165–78. 10.1071/RD9961165 8981641

[73] Caballero I Vazquez JM Centurion F Rodriguez-Martinez H Parrilla I Roca J. Comparative effects of autologous and homologous seminal plasma on the viability of largely extended boar spermatozoa. Reprod Domest Anim. (2004) 39:370–5. 10.1111/j.1439-0531.2004.00530.x 15367272

[74] Bailey JL Bilodeau JF Cormier N. Semen cryopreservation in domestic animals: a damaging and capacitating phenomenon. J Androl. (2000) 21:1–7. 10670514

[75] Parrilla I Vázquez JM Cuello C Gil MA Roca J Di Berardino D. Hoechst 33342 stain and u.v. laser exposure do not induce genotoxic effects in flow-sorted boar spermatozoa. Reproduction. (2004) 128:615–21. 10.1530/rep.1.00288 15509707

[76] Vazquez JM Martinez EA Parrilla I Gil MA Lucas X Roca J. Motility characteristics and fertilizing capacity of boar spermatozoa stained with hoechst 33342. Reprod Domest Anim. (2002) 37:369–74. 10.1046/j.1439-0531.2002.00387.x 12464077

[77] Guthrie HD Johnson LA Garrett WM Welch GR Dobrinsky JR. Flow cytometric sperm sorting: effects of varying laser power on embryo development in swine. Mol Reprod Dev. (2002) 61:87–92. 10.1002/mrd.1134 11774379

[78] Suh TK Schenk JL Seidel GE. High pressure flow cytometric sorting damages sperm. Theriogenology. (2005) 64:1035–48. 10.1016/j.theriogenology.2005.02.002 16125550

[79] Garner DL Evans KM Seidel GE. Sex-sorting sperm using flow cytometry/cell sorting. Methods Mol Biol. (2013) 927:279–95. 10.1007/978-1-62703-038-0_26 22992923

[80] Anel-Lopez L Garcia-Alvarez O Maroto-Morales A Tarantini T Del OD Ortiz JA. Optimization of protocols for Iberian red deer (Cervus elaphus hispanicus) sperm handling before sex sorting by flow cytometry. Theriogenology. (2017) 92:129–36. 10.1016/j.theriogenology.2017.01.023 28237327

[81] Umezu K Hiradate Y Numabe T Hara K Tanemura K. Effects on glycocalyx structures of frozen-thawed bovine sperm induced by flow cytometry and artificial capacitation. J Reprod Dev. (2017) 63:473–80. 10.1262/jrd.2017-065 28701622 PMC5649096

[82] Qin Y Yang S Xu J Xia C Li X An L. Deep insemination with sex- sorted cashmere goat sperm processed in the presence of antioxidants. Reprod Domest Anim. (2018) 53:11–9. 10.1111/rda.13045 29205543

[83] Fantinati P Zannoni A Bernardini C Webster N Lavitrano M Forni M. Laparoscopic insemination technique with low numbers of spermatozoa in superovulated prepuberal gilts for biotechnological application. Theriogenology. (2005) 63:806–17. 10.1016/j.theriogenology.2004.05.005 15629799

[84] Del OD Parrilla I Sanchez-Osorio J Gomis J Angel MA Tarantini T. Successful laparoscopic insemination with a very low number of flow cytometrically sorted boar sperm in field conditions. Theriogenology. (2014) 81:315–20. 10.1016/j.theriogenology.2013.09.031 24157229

[85] Bennett D Boyse EA. Sex ratio in progeny of mice inseminated with sperm treated with H-Y antiserum. Nature. (1973) 246:308–9. 10.1038/246308a0 4586316

[86] Zavos PM Preconception sex determination via intra-vaginal administration of H-Y antisera in rabbits. Elsevier. (1983) 20:235–40. 10.1016/0093-691X(83)90219-4

[87] Bradley MP. Immunological sexing of mammalian semen: current status and future options. Elsevier. (1989) 72:3372–80. 10.3168/jds.S0022-0302(89)79500-X 2697721

[88] Kawarasaki T Welch GR Long CR Yoshida M Johnson LA. Verification of flow cytometorically-sorted X- and Y-bearing porcine spermatozoa and reanalysis of spermatozoa for DNA content using the fluorescence *in situ* hybridization (FISH) technique. Theriogenology. (1998) 50:625–35. 10.1016/S0093-691X(98)00167-8 10732153

[89] Umehara T Tsujita N Shimada M. Activation of Toll-like receptor 7/8 encoded by the X chromosome alters sperm motility and provides a novel simple technology for sexing sperm. PLoS Biol. (2019) 17:e3000398. 10.1371/journal.pbio.3000398 31408454 PMC6691984

[90] Miller DJ. Review: the epic journey of sperm through the female reproductive tract. Animal. (2018) 12:s110–20. 10.1017/S1751731118000526 29551099 PMC9556260

[91] Rickard JP Pool KR Druart X de Graaf SP. The fate of spermatozoa in the female reproductive tract: a comparative review. Theriogenology. (2019) 137:104–12. 10.1016/j.theriogenology.2019.05.044 31230704

[92] Almiñana C Caballero I Heath PR Maleki-Dizaji S Parrilla I Cuello C. The battle of the sexes starts in the oviduct: modulation of oviductal transcriptome by X and Y-bearing spermatozoa. BMC Genomics. (2014) 15:293. 10.1186/1471-2164-15-293 24886317 PMC4035082

[93] Gutierrez-Adan A Perez-Garnelo J Granados J Garde JJ Perez-Guzman M Pintado B. Relationship between sex ratio and time of insemination according to both time of ovulation and maturational state of oocyte. Zygote. (1999) 7:37–43. 10.1017/S0967199499000374 10216915

[94] Dominko T First NL. Relationship between the maturational state of oocytes at the time of insemination and sex ratio of subsequent early bovine embryos. Theriogenology. (1997) 47:1041–50. 10.1016/S0093-691X(97)00061-7 16728054

[95] Grant VJ Irwin RJ. Follicular fluid steroid levels and subsequent sex of bovine embryos. J Exp Zool A Comp Exp Biol. (2005) 303A:1120–5. 10.1002/jez.a.233 16254922

[96] Grant VJ Irwin RJ Standley NT Shelling AN Chamley LW. Sex of bovine embryos may be related to mothers' preovulatory follicular testosterone. Biol Reprod. (2008) 78:812–5. 10.1095/biolreprod.107.066050 18184920

[97] Zhou J Chen L Li J Li H Hong Z Xie M. The semen pH affects sperm motility and capacitation. PLoS ONE. (2015) 10:e132974. 10.1371/journal.pone.0132974 26173069 PMC4501804

[98] Raval NP Shah TM George L Joshi CG. Effect of the pH in the enrichment of X or Y sex chromosome-bearing sperm in bovine. Vet World. (2019) 12:1299–303. 10.14202/vetworld.2019.1299-1303 31641311 PMC6755399

[99] Yu L Zheng Y Feng Y Ma F. Role of L-selectin on leukocytes in the binding of sialic acids on sperm surface during the phagocytosis of sperm in female reproductive tract. Med Hypotheses. (2018) 120:4–6. 10.1016/j.mehy.2018.08.008 30220337

[100] Al-Dossary AA Strehler EE Martin-Deleon PA. Expression and secretion of plasma membrane Ca2+-ATPase 4a (PMCA4a) during murine estrus: association with oviductal exosomes and uptake in sperm. PLoS ONE. (2013) 8:e80181. 10.1371/journal.pone.0080181 24244642 PMC3828235

[101] Osycka-Salut CE Martinez-Leon E Gervasi MG Castellano L Davio C Chiarante N. Fibronectin induces capacitation-associated events through the endocannabinoid system in bull sperm. Theriogenology. (2020) 153:91–101. 10.1016/j.theriogenology.2020.04.031 32447096

[102] Teijeiro JM Cabada MO Marini PE. Sperm binding glycoprotein (SBG) produces calcium and bicarbonate dependent alteration of acrosome morphology and protein tyrosine phosphorylation on boar sperm. J Cell Biochem. (2008) 103:1413–23. 10.1002/jcb.21524 17786920

[103] Mara L Pilichi S Sanna A Accardo C Chessa B Chessa F. Sexing of *in vitro* produced ovine embryos by duplex PCR. Mol Reprod Dev. (2004) 69:35–42. 10.1002/mrd.20147 15278902

[104] Descoteaux L Gnemmi G Colloton J. Ultrasonography of the bovine female genital tract. Vet Clin North Am Food Anim Pract. (2009) 25:733–52. 10.1016/j.cvfa.2009.07.009 19825441

[105] Meagher S Davison G. Early second-trimester determination of fetal gender by ultrasound. Ultrasound Obstet Gynecol. (1996) 8:322–4. 10.1046/j.1469-0705.1996.08050322.x 8978005

[106] Beal WE Perry RC Corah LR. The use of ultrasound in monitoring reproductive physiology of beef cattle. J Anim Sci. (1992) 70:924–9. 10.2527/1992.703924x 1564011

[107] Aurich C Schneider J. Sex determination in horses - current status and future perspectives. Anim Reprod Sci. (2014) 146:34–41. 10.1016/j.anireprosci.2014.01.014 24598214

[108] Erdogan G. Ultrasonic assessment during pregnancy in goats - a review. Reprod Domest Anim. (2012) 47:157–63. 10.1111/j.1439-0531.2011.01873.x 21771111

[109] Dar S Lazer T Shah PS Librach CL. Neonatal outcomes among singleton births after blastocyst versus cleavage stage embryo transfer: a systematic review and meta-analysis. Hum Reprod Update. (2014) 20:439–48. 10.1093/humupd/dmu001 24480786

[110] Williams TJ. A technique for sexing mouse embryos by a visual colorimetric assay of the X-linked enzyme, glucose 6-phosphate dehydrogenase. Theriogenology. (1986) 25:733–9. 10.1016/0093-691X(86)90131-7 16726164

[111] Goldberg EH. H-Y antigen and sex determination. Philos Trans R Soc Lond B Biol Sci. (1988) 322:73–81. 10.1098/rstb.1988.0115 2907805

[112] Wachtel S Nakamura D Wachtel G Felton W Kent M Jaswaney V. Sex selection with monoclonal H-Y antibody. Fertil Steril. (1988) 50:355–60. 10.1016/S0015-0282(16)60086-9 3165072

[113] Singh EL Hare WCD The feasibility of sexing bovine morula stage embryos prior to embryo transfer. Theriogenology. (1980) 14:421–7. 10.1016/0093-691X(80)90053-9

[114] Gardner RL Edwards RG. Control of the sex ratio at full term in the rabbit by transferring sexed blastocysts. Nature. (1968) 218:346–9. 10.1038/218346a0 5649672

[115] Gokulakrishnan P Kumar RR Sharma BD Mendiratta SK Sharma D. Sex determination of cattle meat by polymerase chain reaction amplification of the DEAD box protein (DDX3X/DDX3Y) gene. Asian-Aus J Anim Sci. (2012) 25:733–7. 10.5713/ajas.2012.12003 25049620 PMC4093110

[116] Aasen E Medrano JF. Amplification of the ZFY and ZFX genes for sex identification in humans, cattle, sheep and goats. Biotechnology. (1990) 8:1279–81. 10.1038/nbt1290-1279 1369448

[117] Pomp D Good BA Geisert RD Corbin CJ Conley AJ. Sex identification in mammals with polymerase chain reaction and its use to examine sex effects on diameter of day-10 or−11 pig embryos. J Anim Sci. (1995) 73:1408–15. 10.2527/1995.7351408x 7665371

[118] Bondioli KR Ellis SB Pryor JH Williams MW Harpold MM The use of male-specific chromosomal DNA fragments to determine the sex of bovine preimplantation embryos. Elsevier. (1989) 31:95–104. 10.1016/0093-691X(89)90567-0

[119] Lavery S. Preimplantation genetic diagnosis: new reproductive options for carriers of haemophilia. Haemophilia. (2004) 10:126–32. 10.1111/j.1365-2516.2004.01042.x 15479385

[120] Chrenek P Boulanger L Heyman Y Uhrin P Laurincik J Bulla J. Sexing and multiple genotype analysis from a single cell of bovine embryo. Theriogenology. (2001) 55:1071–81. 10.1016/S0093-691X(01)00467-8 11322235

[121] Herr CM Reed KC Micronanipulation of bovine embryos for sex determination. Elsevier. (1991) 35:45–54. 10.1016/0093-691X(91)90147-6

[122] Tienan Z Jichang L Chengwu L Guicheng H A multiplex PCR assay for the detection of pathogenic genes of EPEC, ETEC and EIEC. J Northeast Agric Univ. (2006) 13:51–4.

[123] Cha RS Zarbl H Keohavong P Thilly WG. Mismatch amplification mutation assay (MAMA): application to the c-H-ras gene. Genome Res. (1992) 2:14–20. 10.1101/gr.2.1.14 1490171

[124] Tavares KCS Carneiro IS Rios DB Feltrin C Ribeiro AKC Gaudencio-Neto S. A fast and simple method for the polymerase chain reaction-based sexing of livestock embryos. Genet Mol Res. (2016) 15:gmr.15017476. 10.4238/gmr.15017476 27050974

[125] Blanes MS Tsoi SCM Dyck MK. Accurate and phenol free DNA sexing of day 30 porcine embryos by PCR. J Visual Exp. (2016) 14:53301. 10.3791/53301 26966900 PMC4828158

[126] Saberivand A Ahsan S. Sex determination of ovine embryos by SRY and amelogenin (AMEL) genes using maternal circulating cell free DNA. Anim Reprod Sci. (2016) 164:9–13. 10.1016/j.anireprosci.2015.10.011 26651950

[127] Yonekawa T Notomi T Watanabe K Masubuchi H Okayama H Amino N. Loop-mediated isothermal amplification of DNA. Nucleic Acids Res. (2000) 28:E63. 10.1093/nar/28.12.e63 10871386 PMC102748

[128] Khamlor T Pongpiachan P Parnpai R Punyawai K Sangsritavong S Chokesajjawatee N. Bovine embryo sex determination by multiplex loop-mediated isothermal amplification. Theriogenology. (2015) 83:891–6. 10.1016/j.theriogenology.2014.11.025 25542460

[129] Almasi MA Almasi G. Loop mediated isothermal amplification (LAMP) for embryo sex determination in pregnant women at eight weeks of pregnancy. J Reprod Infertil. (2017) 18:197–204. 28377900 PMC5359858

